# Sociopolitical Approach to the Launch History of the KBO League: Application of Complex System Paradigm

**DOI:** 10.3390/ijerph18105471

**Published:** 2021-05-20

**Authors:** Won-Chul Bing

**Affiliations:** Division of Sports Science, Baekseok University, Cheonan-si 31065, Korea; bing7@bu.ac.kr

**Keywords:** sport politics, Korean sports culture, historical analysis, professional baseball

## Abstract

The Korean Baseball Organization (KBO) League is a sports culture that Koreans love and enjoy most. However, the launch of the KBO League is related to political issues in Korea. The purpose of this study is to explain the launch history of the KBO league through a sociopolitical approach. The history of the KBO league was explained by applying a complex paradigm that explains sociocultural phenomena from a new perspective. This study used historical analysis, a qualitative study approach. Literature related to Korean professional baseball, complex system theory, sports, and politics were reviewed. This study introduces the characteristics and theory of the complex system paradigm and analyzes the history of the KBO League based on this theory. The edge of chaos, bifurcation point, positive feedback, emergence of the theory of complex system are used as elements of an overall theoretical framework to analyze the history and development of the KBO league. The study results are explained in four frameworks. First, the KBO was launched on the edge of chaos, or in the midst of social chaos provoked by Chun Doo-Hwan, who seized power through a military coup. Second, the Chun Doo-Hwan regime launched the professional baseball league to divert the public’s attention from politics to sports and provided support to construct baseball fields as venues for the national pastime. The Chun Doo-Hwan regime’s appeasement policy became a bifurcation point, which promoted the launch of the professional baseball league. Third, from the viewpoint of the complex system paradigm, the launch of the Korean baseball league was enabled by the positive feedback of the Korea professional baseball promotion committee, established in 1975 under the initiative of Korean American businessman Hong Yoon-Hee. Fourth, the Korean professional baseball league led to the emergence of the consumption culture of professional sports, and it became a national leisure and a crucial part of Korea’s sports culture. In terms of sociopolitical, the KBO League started in the dark of Korean society, but it is becoming a vitality for Korean sports culture and health.

## 1. Introduction

In modern times, countries have actively organized sports. For example, many state governments have hosted sports competitions to unite people from around the world. With the catchphrase of “sports that provide dream and hope to children, romance to the youth, and a pastime activity for the people”, the Korean professional baseball league was launched on March 27, 1982, with the establishment of six professional teams; subsequently increased to 10. In 2019, the Korean Baseball Organization (KBO) League surpassed an accumulated number of 162 million spectators, and it has become a major part of Korea’s national leisure and sports culture [1].

In South Korea, during elections, it is common for both presidential and National Assembly candidates to pose for photos wearing baseball uniforms and swinging bats. In addition, when the postseason of the KBO League starts, open-air channels broadcast baseball games instead of soap dramas, entertainment programs, or news. This demonstrates the profound impact of professional baseball in Korea [2].

It is a significantly meaningful historical work to analyze how the Korean professional baseball league started and has evolved. In prior studies, European and American history scholars have paid keen attention to the history of professional sports games [3,4,5,6]. For example, the inception of the English Premier League has been linked to the formation of the working-class culture. The Spanish professional soccer league began as a symbol of the political resistance and oppression that intensified between regions along the border after the Franco regime. The professional baseball leagues in the United States have evolved into a tool for integrating its multi-racial and multi-ethnic society into a unified national identity [7]. The question of “How did a professional sports league start in a country?” can be considered a key factor capable of explaining several problems regarding the political, cultural, and economic history of that country [8].

The existing studies on Korean professional baseball have focused on the foundational background and developmental process of Korean professional baseball [9], the historical meaning of the launch of the KBO League [10], the life of a professional baseball administrator [11], and the contracts of professional baseball players [12]. In addition, Kim [13] published the following information about professional baseball in Korea: origins, causes, consequences, and implications. Furthermore, Cho [14] investigated governmentality in the relationships between local governments and global sports by examining the contexts of the broadcasting of Major League Baseball (MLB) in South Korea. These previous studies analyzed the historical and developmental processes of the Korean professional baseball league based on literature reviews and empirical studies. However, when the authors of the current study discovered the complex system paradigm, a new perspective for seeing the world, we intuitively grasped the possible existence of a certain order and principle in the historical background and causes that led to the launch of the KBO League, thus representing a different view from the existing historical and social scientific analyses [15].

A paradigm provides scientists with not only a problem that should be explored, but also the way on how to find the solution. If the existing pattern fails to solve the problem, it will be replaced with an alternative. This is so called a paradigm shift. A new paradigm comes with a scientific revolution that replaces the old way [16]. According to Cynarski [17], the paradigm is finally systemic and holistic.

Sociology is traced back to modern times when people start to express their responses to social changes. A social study explores consequences arising from social changes. Over time, a skepticism among sociologists has emerged about explaining such a complex society only based on some grand theories, such as the paradigm of structural functionalism or symbolic interactionism. Luhmann has regarded the lack of universal theories that could be adopted to explain social phenomena as the main cause of a crisis in social science. He was interested in developing a universal social theory making up for shortcomings of many functionalisms including Talcott Parsons’s structural functionalism. Luhmann understood all things in the world under the ‘systems.’ According to him, the world is made of systems. Luhmann’s theory of society assumes that the “modern” society is characterized by the process of functional differentiation [18].

How about sports as a social system? Karl Heinrich Bette has inspected sport elements as one social system. Bette stated that “Sport is a social system with complexity which is highly organized and cannot be arbitrarily transformed.” He stresses that there is a strong need to ensure interdependence and independence of sport because sport is more complex than other social systems in terms of function, value, and structure [19].

Bette [5] also stated that an external image or structure presented by sport and all other things affecting sport cannot be truly understood unless you consider environmental factors of the sport system. He emphasizes that any contradictory function of sport occurring in modern society is related to diverse subfunctions. Bette, in this context, argues that an analysis on the relationship between sport and social systems is necessary in order to understand the modern sport with various interests in relation to these systems [20].

Thus, this study focuses on exploring and explaining the history of the Korean professional baseball industry in a sociopolitical point of view using the ‘complexity paradigm’. Our intuition was founded on the complex system studies critical of the Newtonian mechanical global view and the Darwinian paradigm of reductionism [21,22,23,24], and these confirm that there is a hidden order of systems behind a world intricately intertwined through interactions between an infinite number of parts, objects, and elements, which can help facilitate the understanding and analysis of the world.

In the late 19th century, something emerged that could not be explained by Newtonian thinking regarding diverse natural and social phenomena. With the appearance of non-linear and abnormal phenomena, which could not be explained by linear causality, factors that had previously been neglected had to be reconsidered. Many natural and social phenomena cannot be explained through traditional reductionism alone because they are of a complex and non-linear nature, rather than a linear or mechanical nature [24,25]. We are surrounded by a myriad of natural and social phenomena that are seemingly too complex and chaotic to be defined as a certain order or rule. However, not all of these phenomena are beyond the capacity of human perception [26]. The complex system paradigm posits that with closer examination, we can identify a certain order or rule in such complicated phenomena, and the theory of this order is called the “complex system theory” [27]. Thus, it is meaningful to investigate the process of the launch and development of the KBO League, which has been established as a national leisure and sports pastime, based on the theory of complex system.

Following from their formation in the research field of explaining natural phenomena, studies using the complex system paradigm are now expanding to other fields of interpreting social phenomenon such as financial markets, transportation, communication, and education [28,29,30]. However, only a few studies have analyzed sports-related social phenomena using the complex system paradigm. The first study on sports-related social phenomena using complex system paradigm was a study examining the street-cheering phenomenon in Korea during the 2002 FIFA World Cup Korea/Japan [31].

Research analyzing sports-related phenomena and history using the complex system paradigm is a new attempt that goes beyond the existing frameworks of social, historical, and anthropological study methods [32]. Therefore, this study is expected to provide a new perspective for interpreting the social phenomenon and history of sports and to prompt a variety of follow-up studies. In particular, examining the launch history of the Korea Baseball League using the complex system paradigm will provide an opportunity to discover the hidden order lying behind the launch and development process of professional sports.

The research procedure introduces the characteristics of the complex systems paradigm and previous studies, and analyzes the launch history of KBO League through complex systems theory.

The purpose of this study is to reinterpret the history of KBO League by applying a complex paradigm with a sociopolitical approach. Therefore, we expect our findings to provide a new perspective on the history of the Korean professional baseball league.

This study is the first to analyze the KBO League’s launch history in terms of a complex system paradigm.

## 2. Theoretical Background

### 2.1. Concept of Complex System Paradigm

The theory of complex system starts from the assumption that a natural and social phenomenon is too complex to predict, where a complicated world refers to the complexity of an orderly situation rather than a confusing and tangled situation. The words ‘confusion’ and ‘disorder’ mean ‘chaos’, a hidden order or rule behind the confusion and disorder. The theory of complex system identifies a hidden order based on the premise that a certain unknown order may exist in a chaotic state of nature and society. The theory of complex system perceives either natural or social phenomena as one system, called an ‘open system,’ which is the summation of individuals and populations constantly exchanging energy, as well as communicating and interacting with the external world [33].

Globally renowned scholars have defined the complex system in the following ways: Gell-Mann, a physicist who received the Nobel Prize for Physics in 1969, said, “The feature of the complex system is that the understanding of components only cannot explain the whole completely. The complex system consists of numerous parts, objects and players which are intertwined through interaction [22].” Meanwhile, Arthur [21], an economics professor at the Santa Fe Institute, said, “The complex system is the one in which an infinite number of elements form a certain pattern and have unpredicted properties through mutual interference, and in which each pattern is fed back to each element. The complex system is the system in the process of constant evolution over the course of time.” Finally, Singer [24], a professor of psychology at the Yale School of Medicine, said, “As the complex system involves numerous active actors, it requires comprehensive understanding of their collective behaviors. Such aggregate behavior is nonlinear, hence it cannot be simply explained from summation of the individual component’s behaviors.” To summarize, a complex system is referred to as a system in which mutual interaction of the components comprising the whole results in a new complex phenomenon that differs from the features of the individual components and, thus, another order appears in that phenomenon.

### 2.2. Theory of Complex System and Related Sports Research

The complex system perspective that was first recognized as an independent paradigm approximately 40 years ago arose from various efforts from the late 19th century to seek a new alternative to view the world from various perspectives, and from criticisms of reductionism that had been well established as a methodology for scientific development. The complex system in natural sciences, such as physics, biology, and mathematics, was recognized in the early 20th century, and several theories were developed to investigate the theory of complex system [34]. The theory of complex system began with research aiming to reveal phenomena in natural sciences, and it has developed into research for interpreting the complexity in the social, economic, and historical phenomena of our daily lives. In other words, complex phenomena in our society are intrinsically similar to the complexity of natural phenomena.

The emergence of a system change or a new order in a complex system occurs when it is located on the edge of chaos that is full of uncertainties. Exploration of the principles of living organisms that evolve through adaption to their changing environments has revealed that they can adapt better in an intermediate state than in a stable balance state or a confusion state [35]. The edge of chaos is not a state of stable balance or confusion, but rather an intermediate state between the two conditions, or the state of being internalized with the possibility for unlimited change. In the stable balance state, a small change quickly returns to equilibrium. In a confusion state, a small change is not differentiated, but is instead buried [36]. The edge of chaos is a term that symbolizes the complexity of science, while referring to the creation of a new order on the edge of chaos.

In a complex system, a new system is emerging in a stable order through the chaos phenomenon. During this emergence, the process passes through a crucial moment, called the critical point or bifurcation point, at which time the system undergoes changes that are called the critical phenomenon. As the system nears the critical point, it grows and evolves by passing through the critical point and creating a new order [37].

This chapter reviews the research papers that have applied the complex system paradigm in the sports field. In the field of sports medicine, Bittencourt et al. [38] published a Complex Systems Approach for Sports Injuries. The aim of this paper was to propose a complex system model for sports injuries and to demonstrate how the implementation of complex system thinking may allow us to better address the complex nature of the etiology of sports injuries. In addition, Pol et al. [39] analyzed the relationship between the complex system principle and sports training: “Experiential and scientific knowledge, relating to sports training methodologies, has been historically influenced by reductionist models. Based on complex systems science and theories of biological evolution, we provide a systematization and update of theoretical and methodological principles to transform the understanding of the sports training process” [39]. A study by Gibson and Noakes [40] provided the physiological mechanism of the exercise process. They explained that the exercise process is an example of a complex non-linear dynamic system in which physiological systems interact as part of a complex system to regulate activity before, during, and after the exercise period.

Rubio et al. [41] published a study on complex system theory in team sports using by analyzing a five-on-five basketball contest under the complex systems framework. Their findings guaranteed the dynamics analysis of basketball contests. They proposed that coaches should prepare their teams to be able to analyze environmental information and find new solutions for game constraints. Lebed [42] presented a system approach to games and competitive playing by analyzing three main and interrelated categories: the “game” itself, the “system”, and the “conflict”. He tried to prove that the match (the process of playing the game itself) is a conflict of at least two complex dynamical systems.

Lee and Jang [31] published a case study on the relevance of complexity theory with a focus on the self-organization phenomenon of street cheering in the 2002 World Cup. To summarize their results, first, complexity theory can assist in identifying the activity suggesting that an organization characterized by self-organizational interaction is more effective than one controlled by intended manipulation. Second, complexity theory is a useful concept for explaining a complex social situation that cannot be predicted. Third, in a chaotic society, a dynamic organization of a far-from-equilibrium condition leads to a creative order. It was verified that street cheering in the “2002 World Cup” was closely connected to complexity theory. In short, that study suggested that complexity theory, as understood above, has utility for analyzing both diverse and complicated social phenomena [31].

## 3. Material and Method

This study used historical analysis, a qualitative study approach. To analyze the KBO history, the complexity paradigm was used as a theoretical basis. A total of 78 pieces of study journal articles, books, degree thesis, and newspaper articles were examined regarding the Korea professional baseball, the complexity theory, sports, politics, and others.

## 4. Results

In this chapter, we apply the theory of complex system to investigate the launch of the Korean professional baseball league and its development into a national pastime from the perspective of the complex system paradigm.

### 4.1. Edge of Chaos: The Dismal Korean Society (1979–1982)

**Figure 1:**
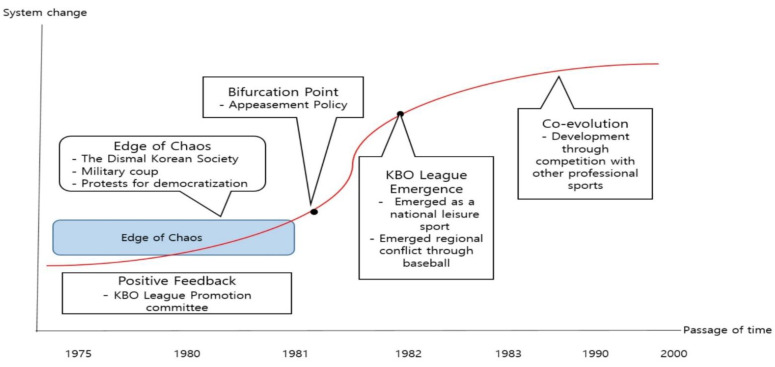
The emergence process of the KBO league from the perspective of the complex system paradigm.

From 1979 to 1982, before the beginning of Korean professional baseball, the Republic of Korea experienced a period of conflict stemming from the conflict between the passion for democracy and the government’s violent oppression. From the perspective of the complex system paradigm (Figure 1), the society was located on the edge of chaos. The edge of chaos is the boundary between order and orderliness, which serves as the starting point for the emergence of transformation, reform, and new order [43].

The military dictatorship (1970s–1980s) was perceived as a dark period for the Republic of Korea [44]. Politically speaking, the revitalizing reforms (Yushin) system (authoritarian governing system that gives the president the authority to rule with an iron fist. It refers to the long-term dictatorship established by President Park Chung-Hee’s declaration of martial law on 17 October 1972) was implemented based on violent oppression against citizens’ basic rights and containment. In addition, two rounds of oil shocks battered economies worldwide.

**Figure 2:**
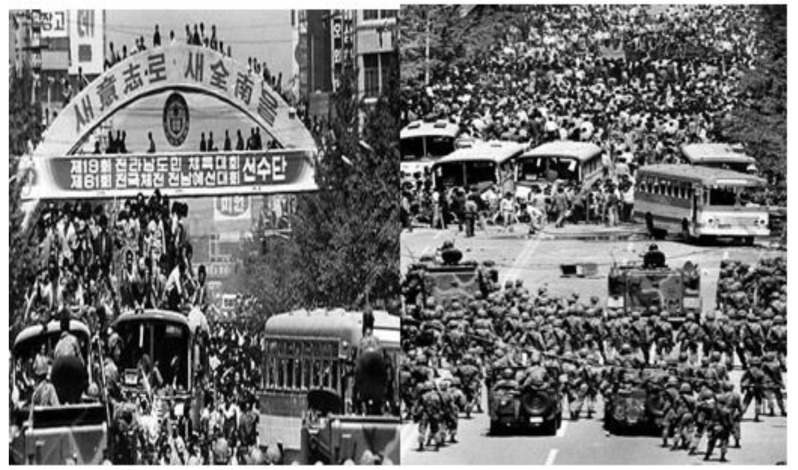
Gwangju Democratic Uprising 18 May 1980–27 May 1980, Data source: 18 May, Democratic Uprising Archives, https://www.518archives.go.kr/, (accessed on 22 January 2021).

The Chun Doo-Hwan regime, which seized power through violent suppression of the 12.12 Military Resurrection (the military revolt on December 12, 1979, led by Chun Doo-Hwan and Roh Tae Woo, who were the leaders of the military) and the May 18 Gwangju Democratic Uprising (the 18-year-long military government was toppled by the assassination of Park Chung-Hee on October 18, 1979. However, the Korean people’s desire for democracy was frustrated by Chun Doo-Hwan’s military coup on December 12, 1979. In May 1980, a massive democratization movement occurred across the country, and the new military regime responded to it by expanding the emergency martial law. In particular, the bloodshed of the oppression by the military regime in Gwangju, South Jeolla Province, sparked a nationwide democratization movement in the 1980s that provided the decisive momentum for the extension of human rights and the development of democracy in Korea) (Figure 2), implemented the 3S (screen, sex, and sports) policy to dispel the public’s discontent with the prolonged dictatorship through initiatives, such as professional baseball [13].

The Chun Doo-Hwan regime, which assumed power through a military coup, faced a political challenge in concealing its own illegitimacy. In September 1981, the coup regime won the bid to host the 1988 Olympic Games and hosted the Seoul Asian Games in November 1986. The national projects of hosting large-scale international sports competitions gradually strengthened the status of sports-related policies [7]. The Chun Doo-Hwan regime mobilized the entire nation in preparation for these large-scale international sports events in order to ease political unrest and promote the expression of nationalism among the people through sports. According to Park and Lim [45], the Chun Doo-Hwan regime attempted to secure political legitimacy by establishing elite sports policies.

The professional baseball league was urgently founded by the Chun Doo-Hwan regime. The plan was devised in just three months, and the KBO League and its six professional baseball teams were created in four months. According to Hong [46], in the 1980s, the chaebols had no choice but to obey the dictatorship’s instructions to create teams for various leagues and to provide financial incentives to elite athletes to raise their morale. The inauguration of the professional baseball league was intended to support the hosting of one-time events and to divert the people’s attention away from politics and toward sports.

In KBO’s first year in 1982, all classes and generations of the Korean people were under substantial pressure. Korean society was on the edge of chaos, as the people were engulfed by issues such as distrust in politics, political oppression, economic instability, democracy movements, and social unrest. Amid these situations, the Korean professional baseball league evolved.

### 4.2. Bifurcation Point: Chun Doo-Hwan Regime’s Appeasement Policy

A fluctuation in the existing order or stability pushes the current system away from equilibrium into a state of non-equilibrium that may threaten the system’s structure such that it can reach the critical point or the bifurcation point. In the complex system paradigm, the components are in a disorderly state when a system collapses from the bifurcation or critical point, after which point they unite to form a new rule and create a new order, when the bifurcation point is reached [47].

Prigogine and Stengers [48] investigated self-organization phenomena by classifying systems into equilibrium state, close-to-equilibrium state, far-from-equilibrium state, and non-equilibrium state in order to identify any pattern of changes that occur at the bifurcation point. They found that if a small fluctuation within the system pushes it far from the equilibrium state, the resulting threat to the system’s structure will induce a bifurcation point that creates an unpredictable condition for a change or innovation.

Because the Chun Doo-Hwan regime seized power in a bloody military coup that suppressed the citizens’ resistance following Park Chung-Hee’s assassination, the regime urgently needed to divert the people’s attention from political matters and to devise a policy to appease the people’s desire for consumption and culture, which had been suppressed during the Park Chung-Hee regime [49].

The Chun Doo-Hwan regime felt they were in a crisis which would lead to the regime being overthrown and the implementation of an appeasement policy unless it suppressed the anger of the people against the regime that oppressed the democratization movement. The regime eased the nightly curfew to allow people to move around freely, liberalized the dress codes of middle and high school uniforms, and eased hairstyle regulations, which were considered the remnants of Japanese colonial rule. In addition, the regime abolished private education to obtain support from students and the underprivileged class, and implemented a series of deregulation measures that included the partial liberalization of overseas travel. In a presidential secretary meeting in May 1981, just after the inauguration of the Fifth Republic of Korea, the creation of professional sports was discussed with an intention to deflect the negative views of the military regime [50]. The touted rationales were involving public sentiment and expanding the use of leisure time, but it was part of the Chun Doo-Hwan regime’s appeasement policy.

The Chun Doo-Hwan regime launched the professional baseball league to divert the people’s attention from politics to sports, and it provided support to make the baseball field a symbol of the national pastime. This appeasement policy became the bifurcation point for the launch of professional baseball [51].

‘Fluctuation’ in the existing order and stability creates a non-equilibrium state by moving the existing system far away from equilibrium. If a non-equilibrium state threatens the structure of the system, it reaches a bifurcation point [52]. This situation, in which the politically oppressed Korean society was on the edge of chaos, met with a bifurcation policy that led to the launch of the KBO.

### 4.3. Positive Feedback: KBO League Promotion Committee

Positive feedback in the complex system is an essential feature in explaining system changes, depending on the sensitivity of the initial conditions. Although most of these changes are weakened by negative feedback, any positive feedback can be rapidly amplified and greatly change the system’s structure to the extent of transforming it into a new system [31]. The emergence of a new system consists of a pattern of specific interactions under an applied feedback or a response to an external stimulus [53].

From the perspective of the complex system paradigm, the launch of Korean professional baseball was possible because of the positive feedback of the Korean professional baseball promotion committee under the initiative of the Korean American businessman Hong Yoon-Hee, who donated $200,000 to promote the creation of the Korean professional baseball league, and persuaded business baseball teams to gradually develop into professional teams [54].

From a historical perspective, many baseball fans say that the professional baseball league was the work of former President Chun Doo-Hwan. However, several years’ worth of efforts by the baseball promotion committee under the initiative of Korean American businessman Hong Yoon-Hee before the launch of the Korean professional baseball league, as well as positive feedback from baseball players and related people, served as the driving forces behind the launch of the KBO League [55].

### 4.4. Emergence: The Emergence of the KBO League as a National Leisure Sport

In a complex system, uncountable components interact with one another to create a new phenomenon or order different from their individual features; this is called ‘emergence.’ A complex system is a system in which emergence occurs. A system that is ‘complex’ is different from one that is merely ‘complicated’, and they can be distinctly differentiated depending on whether or not emergence occurs. Even if a system consists of many components, it is simply a complicated system entangled with many components unless a new major change emerges, in which case it can be called a complex system [56].

The Red Devil syndrome during the 2002 FIFA World Cup Korea/Japan can be explained as an emergence phenomenon. After the Korean War, South Korean people developed an ideological bias against the color red. However, as the media increasingly covered the success of the national football team and supporters’ street cheering during the 2020 World Cup Korea/Japan, football stadiums and street squares came to be crowded with people wearing red T-shirts. As such, a new order that could overcome ideological bias emerged [31].

**Figure 3:**
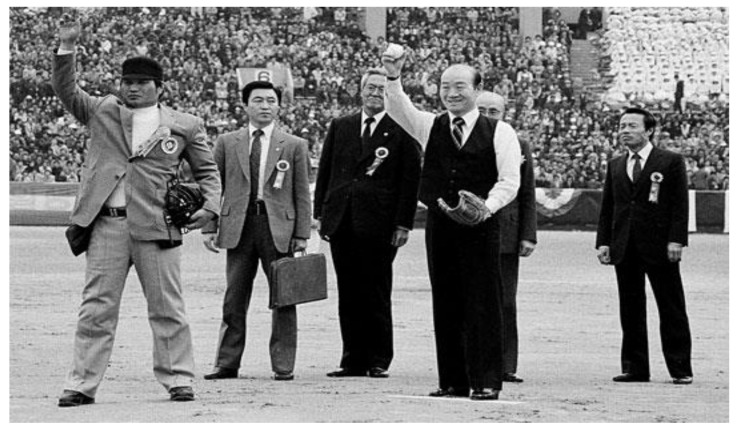
President Chun Doo-Hwan’s first pitch in the professional baseball opening game in 27 March 1982, Data source: real time news media, https://m.ilyo.co.kr/?ac=article_view&entry_id=120011, (accessed on 22 January 2021).

President Chun Doo-Hwan threw the first pitch for the KBO league opening game in March 27, 1982 (Figure 3). Although the KBO league was designed to serve a political purpose for the Chun Doo-Hwan regime, it led to the emergence of the consumption culture of professional sports, and ultimately became a national leisure and a crucial part of Korea’s sports culture. The Korean professional baseball league has grown to become a national sport due to the voluntary participation and consumption of the people, and the number of spectators increased from 1.4 million in 1982 to 3.9 million in 1992, 7.1 million in 2012, and finally 8 million in 2016 [57].

The spread of TV and radio had the greatest influence on the growth of professional baseball fans in the early stage. Professional baseball games and records delivered by media such as TV and radio led to the infinite replication phenomenon of baseball fans. In 1982, after the launch of the KBO League, the Munhwa Broadcasting Corporation (MBC), and Korean Broadcasting System (KBS) regularly broadcasted baseball games. Professional baseball games and records broadcasted by the media attracted people to the baseball fields, thereby increasing fan numbers, even in regions where baseball games were not hosted [58].

The popularity of Korean professional baseball has led to the formation of a unique and creative cheering culture. The enthusiastic cheering of Korean baseball fans has brought on the emergence of an expanding passionate fandom [59]. It is common to see scenes of the KBO League’s zealous cheering culture posted on MLB’s official website. In addition, foreign players have generally evaluated the KBO League’s culture positively. For example, Casey Kelly of the LG Twins noted the ballpark atmosphere and the fans as the greatest advantages of the KBO League. Kelly, who had previously played for the Boston Red Sox and the San Francisco Giants, two popular MLB teams, said, “The biggest advantage of the KBO League is its fandom. Of course, I experienced enthusiastic fans in the Major League Baseball. I received passionate support from fans when I played for Boston and San Francisco. But fans’ emotional attachment is different. American ballparks are generally quiet and spectators shout only at critical moments. But in Korea, people react to every little play [60].” Korean professional baseball fandom is characterized by ‘self-similarity’ and ‘recursiveness’ through cheering songs, synchronized cheering movements, card sections, and waving crowds.

As already discussed above, the Chun Doo-Hwan regime wanted to divert the Korean people’s attention from domestic politics to professional baseball games [13]. However, professional baseball has since established itself as a popular leisure activity [50]. After professional games started being broadcasted live, more people spent time watching the games after work, and professional baseball became a favorite topic of conversation among Koreans. In this way, professional baseball became a cultural phenomenon, as it provided an emotional connection through which people of that time shared their individual everyday lives and their memories of the period.

## 5. Discussion

Historiography on the intersection of sport and politics is a vast field within which six major areas can be identified. (1) German, Italian, Korea and—to a lesser extent—Japanese historians have written extensively about the role of sports under fascist regimes. (2) There have also been numerous efforts to analyze the role of sport in communist societies. (3) Many historians have dealt with sport and the politics of race and ethnicity. (4) European and American historians have also written extensively and with considerable passion about the politics of gender discrimination. (5) The Olympic Games, which their founder intended to be a political force, have been a fifth focus of historical scrutiny. (6) French and German neo-Marxist historians and sociologists have argued that modern sports are a mirror image of capitalist institutions and are, therefore, inherently repressive [61].

According to Dziubiński and Zaczyńska (2019), in authoritarian and totalitarian systems, sport is used in an instrumental way and serves the purposes of a tyrant or regime that is political propaganda and military–utilitarian. In these systems, sport serves to achieve despotic goals and conformistically oriented supporters [62].

Peace-building and nation-building can be achieved through four mechanisms of sport politics: image-building; building a platform for dialogue; trust-building; and reconciliation, integration and anti-racism. Sporting events can be used as a means of building trust between adversaries. On the other, the hostilities between peoples can be mirrored on playing fields [63].

A new emergence phenomenon that occurred after the launch of the KBO was the appearance of regional conflicts stemming from professional baseball games. In 1982, President Chun Doo-Hwan invited the owners of professional baseball teams to a meeting and directly gave them detailed instructions about the expected performance levels of the baseball teams and the cheering culture of spectators, as well as the future operation strategy of the KBO. He emphasized the need to promote the popularity of professional baseball nationwide by leveling off the performance of all teams and emphasizing regional representation, and he asked for the participation of local leaders to support the success of the KBO, as well as for the promotion of a ‘cheering culture’ and the expansion of ‘fandom.’ In particular, the president asked that professional baseball should play a role in the nation’s development by providing pleasure to ‘the people’ and promoting amity with foreign countries. Altogether, the regime that seized power through a military coup hoped to gain the people’s support for the nation and promote unity among the people [64].

However, the KBO, which was launched based on a local franchise system representing specific regions, prompted the emergence of regional conflicts tied to sports. In the first year when the KBO was launched, six professional baseball teams were founded: MBC Chungyong (Seoul), Lotte Giants (Busan), Samsung Lions (Daegu), Haitai Tigers (Gwangju), OB Bears (Daejeon), and Sammi Superstars (Incheon). Baseball matches between the Samsung Lions (Taegu), who represented the Yeongnam region, and the Haitai Tigers (Gwangju), who represented the Honam region, triggered regional conflicts [65].

In 1971, the presidential election race between Park Chung-Hee, who represented the Yeongnam region, and Kim Dae-jung, who represented the Honam region, was the starting point of regional political conflicts. After Park Chung-Hee was elected president, he pursued a development policy in favor of the Yeongnam region, which touched off widespread discontent among people in the Honam region [66]. Therefore, a baseball match between the Samsung Lions from the Yeongnam region and the Haitai Tigers from the Honam region offered an opportunity for people in the Honam region to release their frustration by watching a live broadcast game [67].

In Game 3 of the 1986 Korean Series, the Samsung Lions, the home team, lost to the Haitai Tigers. In response, about 100 Samsung Lions fans set fire to the Haitai Tigers’ team bus, which further inflamed regional conflicts. Professional baseball was no longer simply considered a pastime activity, as it gained a symbolic image that represented regional interactions [68]. The baseball park in Seoul, was a place for the many regional immigrants into the capital city to connect with their hometowns through the different teams that would visit, and in this way, professional baseball fans cheered for their hometown teams and maintained their regional identity. The regional political conflict between Yeongnam and Honam regions continues, as does the regional enmity sparked off by professional baseball.

Studies on the system theory, the mechanism between sports and politics, and sport culture, have been actively conducted in the area of sport humanities [69,70]. A variety of study results have been published in the field of sport humanities to explain the relation between the baseball industry and politics. Lin and Lee (2007) examine the manner in which the Japanese colonial government influenced the development of baseball in Taiwan during the period from 1895 to 1945. The paper explains the role that baseball played in the divided society of this period. It argues that baseball was a political vehicle used by the Japanese colonial government to promote social integration and it evaluates the responses of the Taiwanese to baseball utilized for this purpose [71].

“Pitching Democracy” details how Dominicans used baseball to communicate their expectations for democratic society in their interactions with their government, the United States, other Latin Americans, and each other during the rapid political transitions of the period 1955–1978. “Pitching Democracy” demonstrates Dominicans’ engagement with the ideological debates of the Cold War. Their interactions with baseball representatives from the United States and Cuba influenced the meanings that Dominicans projected onto baseball and democracy [72].

According to Eagleman (2011), racial and nationality-based stereotypes of professional baseball players have been prominent in the U.S. media since the 1800s [73]. Moreover, the 2021 MLB All-Star Game was recently moved out of Atlanta as a result of a Georgia law being passed that restricts voting access in the state. The game ended up being moved to Coors Field in Colorado. In a statement, MLB commissioner Rob Manfred said the decision to move the All-Star Game was “the best way to demonstrate our values as a sport.” The decision was made after consulting with teams, current and former players, and the MLB Players Association [74].

Sports can be manipulated and used as symbols for politics. A nation often uses sports as a means of governing. Companies also try to act on politics utilizing sports. Sports have a powerful symbolism that can be generally misused [75]. The KBO league started as a tool to endorse a military coup government through a symbolic manipulation. Over time, the league wandered away from the political handling and began developing a new culture.

The increased number of professional baseball spectators is the result of co-evolution under mutual influence through competition with other mega sports events, such as the World Cup and the Olympics. However, the Korean professional baseball league experienced moments of crisis. For example, the number of spectators increased to 5.5 million in 1995, but then declined steadily to 2.3 million in 2004. The reasons for the spectators’ decline included external factors, such as dwindling consumer consumption and clubs’ financial difficulties from the 1998 IMF crisis, the football fever after the 2002 Korea–Japan World Cup, internal factors, such as game manipulation scandals, wide performance gap between clubs, and under-performing popular clubs [76].

In Korea, the professional leagues of football and Ssireum (traditional wrestling) started in 1983, followed by basketball in 1997 and volleyball in 2005. The professional golf league started in 1968 amid low public interest, but because of the emergence of world-class Korean golfers, it is enjoying popularity. Korean professional sports leagues are advancing through competition with each other. Football received fervent national support during the World Cup and the Olympic Games, and this fever spread to the domestic professional football league. In particular, the Red Devil’s cheering enthusiasm during the 2002 Japan–Japan World Cup brought an explosion of popularity to the domestic professional football. Moreover, Park Se-Ri’s victory at the 1998 U.S. Women’s Open Championship gave confidence to the Korean people in the dire domestic situation of the IMF crisis [77]. Since then, the KLPGA has gained enormous popularity and has operated a successful league. In the face of these challenges from by other professional sports to its popularity, the Korean professional baseball league stoked the fever of professional baseball by achieving outstanding results at international competitions. The Korean national team, mainly comprising of professional baseball players, won the championship of the 2006 World Baseball Classic (WBC), received acclaim from the people, and won the gold medal at the Beijing Olympics in 2008, which boosted the popularity of the domestic professional baseball league [78]. The co-evolution of the professional baseball league continues to retain the status of a national leisure and sports culture through competition with other professional sports such as soccer, basketball, volleyball, and golf, through marketing competition to promote the league, and by the nurturing of rookie players.

## 6. Conclusions

The purpose of this study was to investigate the characteristics and theory of the complex system paradigm and to analyze the launch history of the KBO league based on this theory. Analyzing the history of the Korean professional baseball league from the perspective of the complex system paradigm, which emerged as a new alternative that recognizes the limitations in understanding the world from the perspective of traditional reductionism, is thought to be a meaningful exercise. This study has presented a new perspective on the launch history of the Korean professional baseball league by moving beyond the traditional sports humanities’ research method and perspective and analyzing the launch history of the KBO League through the lens of the complex system paradigm.

The analysis results of the launch history of the KBO League from the viewpoint of the complex system paradigm are summarized as follows. First, the KBO was launched on the edge of chaos, or in the midst of social chaos provoked by Chun Doo-Hwan, who seized power through a military coup. Many factors in the KBO League’s first year exerted pressure on all classes and generations of the Korean people. Korean society at that time was on the edge of chaos, facing issues, such as distrust in politics, political oppression, economic insecurity, democratization movement, and social unrest, and the KBO evolved in that turbulent situation.

Second, the Chun Doo-Hwan regime engaged in an appeasement policy after it sensed the risk of subversion by a nationwide revolt if it did not calm the people’s anger against the regime’s suppression of the democratization movement. The Chun Doo-Hwan regime launched the professional baseball league to divert the public’s attention from politics to sports and provided support to construct baseball fields as venues for the national pastime. The Chun Doo-Hwan regime’s appeasement policy became a bifurcation point which promoted the launch of the professional baseball league.

Third, from the viewpoint of the complex system paradigm, the launch of the Korean baseball league was enabled by the positive feedback of the Korea professional baseball promotion committee, established in 1975 under the initiative of Korean American businessman Hong Yoon-Hee. Many baseball fans believe that the professional baseball league was the work of Chun Doo-Hwan from a historical perspective. However, many years’ previous efforts by the promotion committee organized under the initiative of Korean American businessman Hong Yoon-Hee prior to the launch of the professional baseball league, as well as positive feedback among baseball players and related people, served as the driving forces behind the launch of the Korean professional baseball league.

Fourth, the Korean professional baseball league led to the emergence of the consumption culture of professional sports, and it became a national leisure and a crucial part of Korea’s sports culture. The dissemination of TV and radio played the most critical role in promoting the professional baseball league at the early stage, along with the support of the media and Korean professional baseball fans’ unified and passionate cheering, which showed the characteristics of ‘self-similarity’ and ‘recursiveness.’

The limitation of this study was that interviews with original members (who were involved in the start of the KBO league) were not performed. A qualitative study about KBO league founders is needed in the future.

## Data Availability

Not applicable.

## References

[1] Choi Y., Kim H. (2011). A statistical study on Korean baseball league games. Korean J. Appl. Stat..

[2] Lee Y.H. (2012). A Study on Korea Professional Baseball Fandom. Ph.D. Dissertation.

[3] Nathan D.A. (2014). Baseball as the National Pastime: A fiction whose time is past. Int. J. Hist. Sport.

[4] Kelly J.D. (2011). Reason and magic in the country of baseball. Int. J. Hist. Sport.

[5] Taylor M. (2015). Parallel Fields: Labour History and Sports History. Int. J. Hist. Sport.

[6] Holt R. (2014). Historians and the History of Sport. Sport Hist..

[7] Kim U.S. (2019). Changes of Korea society in 1970’s and the birth of professional baseball. Acad. Korean Stud..

[8] White G.E. (2014). Creating the National Pastime: Baseball Transforms Itself, 1903–1953.

[9] Kim M.K. (2012). Political backgrounds involved in the foundation of Korea professional baseball. Korean Soc. Sport Anthropol..

[10] Kim J.Y. (2018). The historical meaning of Korean professional baseball launching examined through the players of MBC Blue Dragon team. Korean J. Hist. Phys. Educ. Sport Dance.

[11] Cho J.H. (2018). The life of Lee Yong Il, the midwife of the birth of Korean professional baseball league. Korean J. Hist. Phys. Educ. Sport Dance.

[12] Yoo G.R. (2017). A brief review on the labor law Issues on the contract for professional baseball players. Korean Assoc. Sports Entertain. Law.

[13] Kim B. (2008). Professional baseball in Korea: Origins, causes, consequences and implications. Int. J. Hist. Sport.

[14] Cho Y. (2008). Broadcasting major league baseball as a governmental instrument in South Korea. J. Sport Soc. Issues.

[15] Nourazari S., Lovato K., Weng S.S. (2021). Making the Case for Proactive Strategies to Alleviate Homelessness: A Systems Approach. Int. J. Environ. Res. Public Health.

[16] Kuhn T.S. (2012). The Structure of Scientific Revolutions.

[17] Cynarski W.J. (2014). The New Paradigm of Science Suitable for the 21st Century. Procedia Soc. Behav. Sci..

[18] Luhmann N. (1995). Social Systems.

[19] Bette K. (1999). Systemtheorie und Sport.

[20] Bette K. (2015). Sportsoziologie.

[21] Arthur W.B. (1999). Complexity and the economy. Science.

[22] Gell-Mann M. (1995). The Quark and the Jaguar: Adventures in the Simple and Complex.

[23] Whitesides G.M., Ismagilov R.F. (1999). Complexity in chemistry. Science.

[24] Singer J.L., Morowitz H., Singer J.L. (1995). Mental processes and brain architecture: Confronting the complex adaptive systems of human thought (an overview). The Mind, the Brain, and Complex Adaptive Systems.

[25] Anderson P.W. (1972). More is different. Science.

[26] Manson S.M. (2001). Simplifying Complexity: A review of complexity theory. Geoforum.

[27] Samsung Economic Research Institute (1997). Understanding and Application of Complexity Science.

[28] Siegenfeld A.F., Bar-Yam Y. (2020). An introduction to complex systems science and its applications. Complexity.

[29] Aydinoglu A.U. (2013). Toward a new understanding of virtual research collaborations: Complex adaptive systems framework. Sage Open.

[30] Jacobson M.J., Levin J.A., Kapur M. (2019). Education as a complex system: Conceptual and methodological implications. Educ. Res..

[31] Lee G.M., Jang S.H. (2004). A case study on the relevance of complexity theory: On the focus of self-organization phenomenon of street cheer in the 2002 the World Cup. Korean Soc. Public Adm..

[32] Smith A. (2004). Complexity theory and change management in sport organizations. EMERGENCE-MAHWAH-LAWRENCE ERLBAUM-.

[33] von Bertalanffy L. (1969). General System Theory: Foundations, Development, Applications.

[34] Yoon Y.S., Chae S.B. (2005). Introduction to Complex System.

[35] Kauffman S.A., Kuk T.Y. (2002). Edge of Chaos.

[36] Yang Y.J. (2007). Importance of Initial Conditions in Organizational Change from a Complex Adaptive System Perspective. Master’s Thesis.

[37] Kim Y.U. (1999). Flap of Chaos’ Wings.

[38] Bittencourt N.F.N., Meeuwisse W.H., Mendonca L.D., Nettel A., Ocarino J.M., Fonseca S.T. (2016). Complex systems approach for sports injuries: Moving from risk factor identification to injury pattern recognition–narrative review and new concept. Br. J. Sports Med..

[39] Pol R., Balagué N., Ric A., Torrents C., Kiely J., Hristovski R. (2020). Training or Synergizing? Complex Systems Principles Change the Understanding of Sport Processes. Sports Med. Open.

[40] Gibson A.S.C., Noakes T.D. (2004). Evidence for complex system integration and dynamic neural regulation of skeletal muscle recruitment during exercise in humans. Br. J. Sports Med..

[41] Rubio J., Godoy S., Alonso M.C., Medina A. (2013). Complex system theory in team sports, example in 5 on 5 basketball contest. Rev. Psicol. Deporte.

[42] Lebed F. (2006). System approach to games and competitive playing. Eur. J. Sport Sci..

[43] Kauffman S.A. (1993). The Origins of Order: Self-Organization and Selection in Evolution.

[44] Sarantakes N.E., Wickham J.A. (2001). Korea on the Brink: From the “12/12” Incident to the Kwangju Uprising, 1979–1980. Naval War Coll. Rev..

[45] Park J., Lim S. (2015). A chronological review of the development of elite sport policy in South Korea. Asia Pac. J. Sport Soc. Sci..

[46] Hong E. (2011). Elite sport and nation-building in South Korea: South Korea as the dark horse in global elite sport. Int. J. Hist. Sport.

[47] Gao Q., Ma J. (2009). Chaos and Hopf bifurcation of a finance system. Nonlinear Dyn..

[48] Prigogine I., Stengers I. (2018). Order out of Chaos: Man’s New Dialogue with Nature.

[49] Baker E.J. (1982). Politics in South Korea. Curr. Hist. (pre-1986).

[50] Sim Y.J. (2014). Professional baseball policy and ‘National Leisure’ in the period of the Fifth Republic. J. Hist..

[51] Park S.M. (2010). The Paradox of Postcolonial Korean Nationalism: State-Sponsored Cultural Policy in South Korea, 1965–Present. J. Korean Stud..

[52] Arnold V.I., Afrajmovic V.S., Il’yasenko U.S., Shil’nikov L.P. (2013). Bifurcation theory and catastrophe theory. Dynamical Systems V.

[53] Lewin R. (1999). Complexity: Life at the Edge of Chaos.

[54] Hong Y.P. The Secret Story of 40 Years of Professional Baseball, OSEN. http://osen.mt.co.kr/article/G1111510406.

[55] yong Ha W. (1997). Korea Sports in the 1980s and the Seoul Olympic Games of 1988.

[56] Sawyer R.K. (2005). Social Emergence: Societies as Complex Systems.

[57] KBO Audience Status. https://www.koreabaseball.com/History/Crowd/GraphTeam.aspx.

[58] Choi S. (2016). A Study on the Determinants of Fans’ Team Identification in KBO League: Focused on the Effects of Kids Marketing. J. Korea Aca. Ind. Coop. Soc..

[59] Chung J. (2014). An Analysis on the Value of Korea Professional Baseball as TV Contents. J. Korea Contents Assoc..

[60] Sports Seoul. http://www.sportsseoul.com/news/read/883631.

[61] Guttmann A. (2003). Sport, Politics and the Engaged Historian. J. Contemp. Hist..

[62] Dziubiński Z., Zaczyńska vel Zaczek J. (2019). Pozycja sPortu w systemach Politycznych wsPółczesnego świata. Rozprawy Naukowe Akademii Wychowania Fizycznego we Wrocławiu.

[63] Nygård H.M., Gates S. (2013). Soft power at home and abroad: Sport diplomacy, politics and peace-building. Int. Area Stud. Rev..

[64] Kim M.K. (2012). The founding background and process of Korean professional baseball. Sports Anthropol. Res..

[65] Choi C.S., Kim B.S. (2011). The relationship between professional baseball fans’ team attachment, community identity, and cooperative intentions. J. Korean Soc. Sports Soc. Sci..

[66] Lim S.W., Lee K.M. (2003). The Effects of Professional Baseball Games between Youngnam and Honam Teams on Regional Emotion. Korean Soc. Soc. Sport.

[67] Kim H.K., Nam J.S., Bae S.M. (2012). Share Your Memories, Sports Korean History.

[68] Naver Sports News. https://sports.news.naver.com/news.nhn?oid=064&aid=0000002041.

[69] Cynarski W.J. (2015). Way of martial arts and politics. e-Politikon. Kwartalnik Naukowy Ośrodka Analiz Politologicznych Uniwersytetu Warszawskiego.

[70] Lee K., Johnson J.A. (2020). Ambush Marketing in Sport Taekwondo and How to Prevent It. Ido Mov. Cult. J. Martial Arts Anthropol..

[71] Lin C., Lee P. (2007). Sport as a medium of national resistance: Politics and baseball in Taiwan during Japanese colonialism, 1895–1945. null.

[72] Yoder A.R. (2014). Pitching Democracy: Baseball and Politics in the Dominican Republic, 1955–1978. Ph.D. Thesis.

[73] Eagleman A.M. (2011). Stereotypes of Race and Nationality: A Qualitative Analysis of Sport Magazine Coverage of MLB Players. J. Sport Manag..

[74] MLB NEWS ′21 All-Star Game, Draft moved from Atlanta. https://www.mlb.com/news/2021-all-star-game-draft-relocated.

[75] Houlihan B. (1997). Sport, National Identity and Public Policy. Nat. Natl..

[76] Lee K., Yoo B. (2002). A study on how to increase spectators according to attribute evaluation when watching a professional baseball game. J. Korean Sports Ind. Manag. Assoc..

[77] Seoul Shinmun (1998). Park Se Ri’s Conquest of Summit. Seoul Shinmun.

[78] Cho Y. (2016). Toward the post-Westernization of baseball? The national-regional-global nexus of Korean Major League Baseball fans during the 2006 World Baseball Classic. Int. Rev. Soc. Sport.

